# Contrast-induced acute kidney injury and adverse clinical outcomes risk in acute coronary syndrome patients undergoing percutaneous coronary intervention: a meta-analysis

**DOI:** 10.1186/s12882-018-1161-5

**Published:** 2018-12-22

**Authors:** Yi Yang, Kaisha C. George, Ran Luo, Yichun Cheng, Weifeng Shang, Shuwang Ge, Gang Xu

**Affiliations:** 1 Department of Nephrology, Tongji Hospital Affiliated with Tongji Medical College, Huazhong University of Science and Technology, 1095 Jie Fang Avenue, Wuhan, Hubei 430030 People’s Republic of China; 2 Department of Nephrology, Puai Hospital, Tongji Medical College, Huazhong University of Science and Technology, Wuhan, Hubei 430000 People’s Republic of China

**Keywords:** Contrast-induced acute kidney injury, Acute Coronary syndrome, Percutaneous Coronary intervention, Risk, Meta-analysis

## Abstract

**Background:**

Recent studies have shown associations between contrast-induced acute kidney injury (CI-AKI) and increased risk of adverse clinical outcomes in acute coronary syndrome (ACS) patients undergoing percutaneous coronary intervention (PCI); however, the estimates are inconsistent and vary widely. Therefore, this meta-analysis aimed to evaluate the precise associations between CI-AKI and adverse clinical consequences in patients undergoing PCI for ACS.

**Methods:**

EMBASE, PubMed, Web of Science™ and Cochrane Library databases were systematically searched from inception to December 16, 2016 for cohort studies assessing the association between CI-AKI and any adverse clinical outcomes in ACS patients treated with PCI. The results were demonstrated as pooled risk ratios (RRs) with 95% confidence intervals (CI). Heterogeneity was explored by subgroup analyses.

**Results:**

We identified 1857 articles in electronic search, of which 22 (*n* = 32,781) were included. Our meta-analysis revealed that in ACS patients undergoing PCI, CI-AKI significantly increased the risk of adverse clinical outcomes including all-cause mortality (18 studies; *n* = 28,367; RR = 3.16, 95% CI 2.52–3.97; I^2^ = 56.9%), short-term all-cause mortality (9 studies; *n* = 13,895; RR = 5.55, 95% CI 3.53–8.73; I^2^ = 60.1%), major adverse cardiac events (7 studies; *n* = 19,841; RR = 1.49, 95% CI: 1.34–1.65; I^2^ = 0), major adverse cardiovascular and cerebrovascular events (3 studies; *n* = 2768; RR = 1.86, 95% CI: 1.42–2.43; I^2^ = 0) and stent restenosis (3 studies; *n* = 130,678; RR = 1.50, 95% CI: 1.24–1.81; I^2^ = 0), respectively. Subgroup analyses revealed that the studies with prospective cohort design, larger sample size and lower prevalence of CI-AKI might have higher short-term all-cause mortality risk.

**Conclusions:**

CI-AKI may be a prognostic marker of adverse outcomes in ACS patients undergoing PCI. More attention should be paid to the diagnosis and management of CI-AKI.

**Electronic supplementary material:**

The online version of this article (10.1186/s12882-018-1161-5) contains supplementary material, which is available to authorized users.

## Background

Acute coronary syndrome (ACS), including ST-elevation myocardial infarction (STEMI), non-ST elevation myocardial infarction (NSTEMI) and unstable angina (UA), is one of the most dangerous presenting conditions. It usually results from the abrupt reduction of coronary blood flow caused by the ruptured coronary atherosclerotic lesion and superimposed acute thrombosis. Primary percutaneous coronary intervention (PCI) is recommended as a first-line treatment of the reperfusion strategy and it contributes to the salvage of the myocardium and improvement of the prognosis [1–3]. However, even after successful PCI, both the short-and long-term morbidity and mortality of ACS patients undergoing PCI are alarmingly high [4–6]. Therefore, it is critical to investigate the risk factors associated with worse outcomes.

Contrast-induced acute kidney injury (CI-AKI), also known as contrast-induced nephropathy, is a frequent complication of PCI due to the use of iodinated contrast agent [7–10]. In particular, patients who underwent primary PCI are at higher risk of CI-AKI than those treated with elective PCI due to hemodynamic instability and lack of adequate renal prophylactic therapy [8, 11]. The primary manifestation is the mild decline in renal function, occurring 1 to 3 days after contrast media exposure. According to several reports, CI-AKI after PCI for ACS was associated with adverse clinical outcomes, including all-cause mortality [12–14], short-term in-hospital mortality [15–17], major adverse cardiac events (MACE) [18–20], major adverse cardiovascular and cerebrovascular events (MACCE) [21–23] and stent restenosis [19, 24, 25]. However, several other studies demonstrated the inconsistent results [26, 27]. In addition, the evaluated values among studies varied widely. For example, the increased risk of all-cause mortality for CI-AKI patients after PCI for ACS varied from 54% [26] to 799% [17].

The aim of the present meta-analysis was to evaluate the associations between CI-AKI and adverse clinical consequences, including all-cause mortality, short-term all-cause mortality, MACE, MACCE and stent restenosis in patients undergoing PCI for ACS.

## Methods

Our study was designed, conducted and reported based on Preferred.

Reporting Items for Systematic Reviews and Meta-Analyses (PRISMA).

guidelines (see Additional file 1) [28].

### Search strategy

EMBASE, PubMed, Web of Science™ and Cochrane Library databases were systematically searched from inception to December 16, 2016 using Google Chrome software with following terms: “acute myocardial infarction”, “AMI”, “myocardial infarction”, “MI”, “non-ST segment elevation myocardial infarction”, “ST-segment elevation myocardial infarction”, “acute coronary syndrome”, “ACS”, “unstable angina”, “acute ST-elevation myocardial infarction”, “acute non-ST segment elevation myocardial infarction”, “complete revascularization”,“revascularization”, “revascularization”, “percutaneous coronary intervention”, “PCI”, “contrast-induced acute kidney injury”, “contrast-induced nephropathy”, “contrast media use”, “CIN”, “CI-AKI”, “mortality”, “cardiovascular”, “events”, “death”, “outcome”, “Hemodialysis”, “Haemodialysis”, “Dialysis”, “peritoneal dialysis”, “Adverse effect”, “prognosis”, “chronic kidney disease”, “CKD”, “end-stage renal disease”, “ESRD” (see Additional file 2). Furthermore, we searched the reference lists of included articles for additional studies. The full text of a record was reviewed if there was any doubt to the eligibility of it. Two of the authors (Y.Y. and K.G.) independently screened titles and abstracts, analyzed full-text articles, and ascertained the final eligible records. Divergences were resolved by discussion or consulting a third author.

### Selection criteria

We selected articles that (1) were cohort studies published as original studies, (2) enrolled the populations of general PCI-treated ACS patients, including acute myocardial infarction, non ST-segment elevation myocardial infarction, ST-segment elevation myocardial infarction, unstable angina pectoris or ACS (i.e., without any additional pathologies, neither be given other special operation or interventional therapy), (3) observed the exposure of CI-AKI with clear definition, (4) evaluated the associations between CI-AKI and risk of any clinical outcomes during follow-up, and quantitative effect size could be extracted, calculated or obtained from original authors, including relative risks, hazard ratios or odds ratios (RRs, HRs or ORs) and associated 95% confidence intervals (CIs), and (6) had no restriction of language.

### Exclusion criteria

Studies should be excluded by the following criteria: (1) meta-analyses, reviews, editorials, meeting abstracts, comments, letters, notes, animal studies, case-control and cross-sectional studies were excluded; (2) we could not extract available data from the published result, and the authors did not reply our request for more data; (3) only one or two studies evaluated the association between CI-AKI and risk of the same outcome.

If two articles were from the same cohort but reported the different outcomes, we included both; otherwise, we included the most informative study.

### Data extraction

We extracted the information of interest from each study including study characteristics (study group name, publication year, geographical location, study design, sample size, clinical scenario, age at baseline, male percentage, follow-up duration, prevalence of CI-AKI, CI-AKI definition, smoker percentage, body mass index, Killip class, left ventricular ejection fraction, baseline serum creatinine, baseline eGFR, Family history for coronary artery disease), complication (prevalence of hypertension, diabetes, hyperlipemia), diseases’ history (myocardial infarction, previous coronary bypass surgery, PCI), medications for ACS (Aspirin, ACEI/ARB, β-blocker, Statins), use of contrast volume and confounder-adjusted and unadjusted risk estimates (including RRs, HRs and ORs) and 95% CIs for any adverse outcome. All outcomes in our eligible articles included all-cause mortality, in-hospital mortality, 30-day mortality, cardiovascular mortality, MACE, MACCE, stent restenosis, reinfarction and major bleeding. We conducted meta-analyses if more than two studies had available data for an outcome. Two authors independently extracted data using standardized tables and resolved discrepancies through discussion and consensus. We made a request to the corresponding author of original studies for further information when necessary and included it if responses were obtained.

### Quality assessment

We evaluated the quality of studies using Newcastle–Ottawa Quality Assessment Scale (NOS) of cohort studies [29] which assessed the following: (1) exposed cohort truly or somewhat representative, (2) non-exposed cohort drawn from the same community as the exposed cohort, (3) ascertainment of exposure, (4) outcome of interest not present at start, (5) study controls for age, (6) study controls for≥3 additional risk factors, (7) assessment of outcome (independent blind assessment or record linkage), (8) follow-up≥24 m, (9) complete accounting for cohorts or subjects lost to follow-up unlikely to introduce bias. This scale allocates a maximum of 9 points for quality of study participants. Overall study quality was graded as good (score, 7–9), fair (score, 4–6), or poor (score, 0–3) [30]. Two authors performed the quality assessment independently and divergence was resolved by discussion or consensus.

### Statistical analysis

The studies included in our analysis reported different effect measures (HRs, RRs or ORs). The RR was used as a common measure of association across studies. We combined HRs as RRs in our article. OR was transformed into RR according to the formula RR = OR/[(1-P0) + (P0 × OR)] where P0 stands for the incidence of CI-AKI whenever possible [31]. Otherwise, ORs was considered as RRs because of the low incidence of CI-AKI. Heterogeneity of RRs across included studies was examined using χ^2^ based on Q-statistical test and quantified by I^2^ index. Roughly, heterogeneity was considered significant if *P* < 0.10 and Higgins I^2^ values of 25, 50, and 75% were respectively considered as low, moderate, and high inconsistencies. We used random effects models when there was obvious heterogeneity among studies (I^2^ ≥ 50%); otherwise fixed effects models were used. Publication bias was assessed through Begg’s and Egger’s testing (*P* < 0.10 was significant) as well as funnel plot. Jackknife sensitivity analyses were conducted by recalculating pooled RRs with the removal of a single study once from the baseline group. To explore the source of heterogeneity, subgroup meta-regressions were conducted. All analyses and graphs were made using Stata 10.0 (College Station, TX, USA) and GraphPad Prism 6. *P* < 0.05 by 2 tailed were set to be significant.

## Results

### Literature search

**Fig. 1:**
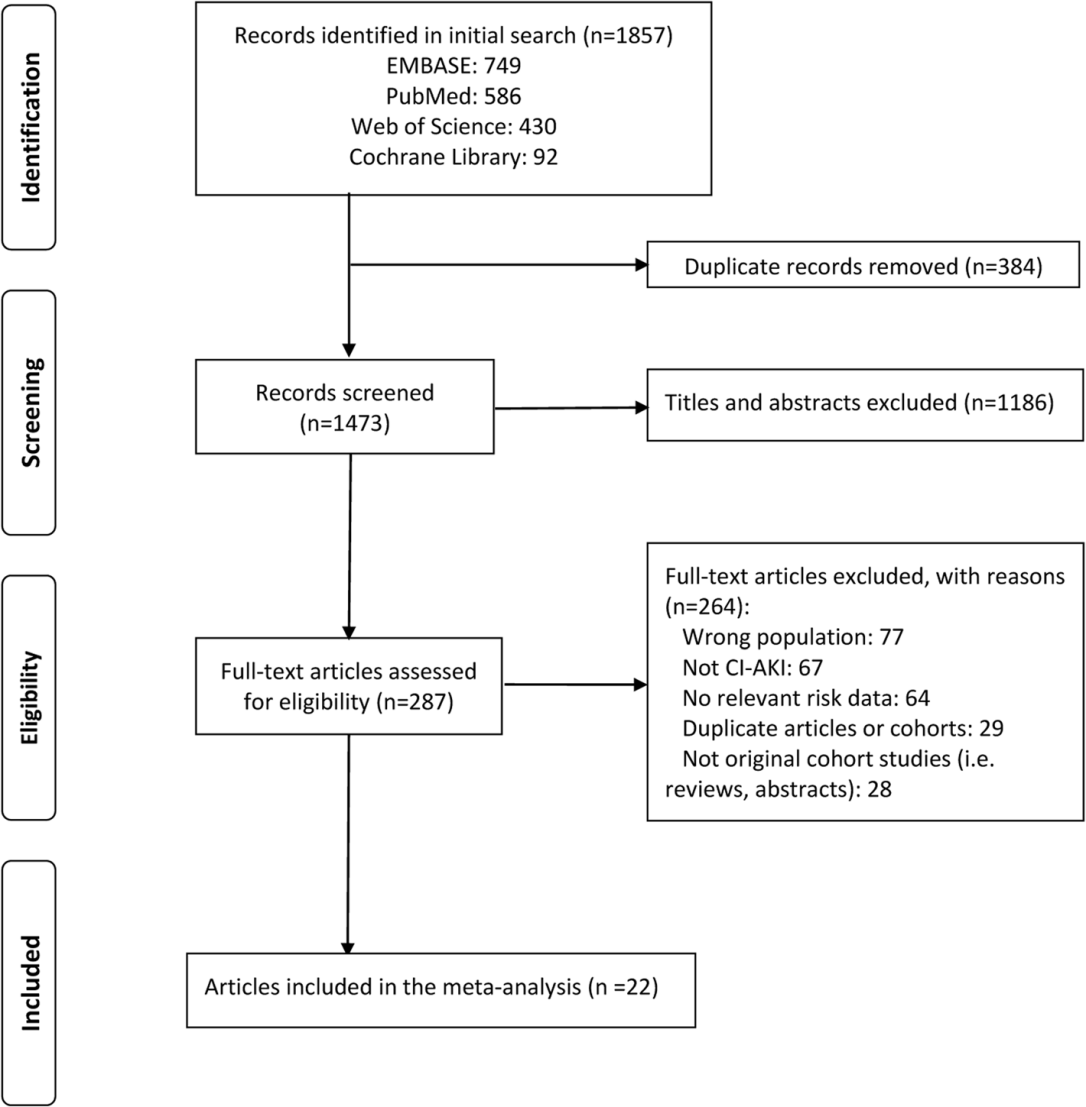
Flow chart of the systematic review. Abbreviations: CI-AKI, contrast-induced acute kidney injury.

We identified 1857 articles in a systematic electronic search, of which 22 [12–27, 32–37] articles were included for the current meta-analysis after detailed evaluation (Fig. 1). No additional article was found through manual search of the reference lists in these studies.

### Study characteristics

Characteristics of the 22 included studies (prospective: 12; retrospective: 10) were summarized in Table 1. Studies were published between 2003 and 2016, and 13 were from Asia, 5 from Europe, 3 from North America and 1 from Australia. The number of participants ranged from 194 to 3822 (32,781 in total, male: 75.9%), and the mean age ranged from 56.4 and 69.0 years across studies. CI-AKI occurred in 4809 patients (14.6%) on average. The definition of CI-AKI varied among the trials. Other relevant characteristics, complications, previous history and medications were shown in Additional files 3 and 4.

**Table 1:** Characteristics of included studies

Study	Country	Study design	Sample size (n)	Clinical scenario	Mean age (y)	Male/%	Follow-Up (m)	CI-AKI (%)	Type of PCI	Contrast volume/ml	CI-AKI Definition (after PCI)
Sadeghi et al. 2003 [32]	USA	PC	2082	AMI	59.3	73.3	Max: 12	4.6	primary	–	IScr ≥0.5 mg/dL within the index hospitalization (5.7 ± 3.5 days)
Uyarel et al. 2009 [37]	Turkey	RC	2521	STEMI	56.5	82.9	Median: 21	25.0	primary	237.0	IScr ≥0.5 mg/dl or 25% within 72 h
Wickenbrock et al. 2009 [12]	Germany	PC	392	STEMI 51.8% NSTEMI 48.2%	64	71.4	During hospitalization	11.5	primary	234.9	IScr > 0.5 mg/dl within 72 h
Akkaya et al. 2011 [13]	Turkey	RC	2486	STEMI	56.4	83.3	Median: 21	24.4	primary	236.3	IScr ≥0.5 mg/dl or 25% within 72 h
Chong et al. 2011 [33]	Singapore	RC	3822	NSTEMI 36.1% UA 63.9%	57.1	78.1	Max: 6	8.4	elective	–	IScr ≥0.5 mg/dl or 25% within 48 h
Wi et al. 2013 [47]	Finland	PC	1041	STEMI 49% NSTEMI 51%	62.7	72.0	Mean: 22.8 Max: 24	14.2	elective	219	IScr > 0.5 mg/dL or 25% within 48 h when no other major kidney insult
Kume et al. 2013 [18]	Japan	RC	194	STEMI	68.1	66.5	Mean: 36	11.9	primary	172.7	IScr > 0.5 mg/dl or 50% within 48 h
Lucreziotti et al. 2014 [14]	Italy	PC	323	STEMI	65.3	71.5	Median: 18 Max: 29.7	15.2	primary	259	IScr ≥0.5 mg/dl or 25% during the first 3 post-procedural days.
Narula et al. 2014 [19]	USA	PC	2968	STEMI	60.4	76.5	Max: 36	16.1	primary	228.2	IScr ≥0.5 mg/dL or 25% within 48 h
Watabe et al. 2014 [22]	Japan	RC	1059	STEMI 56.7% NSTEMI 15.3% UA 28.0%	69.0	76.3	Mean: 14.5	15.5	primary	184.2	IScr ≥0.5 mg/dL or 25% within 1 week
Akin et al. 2015 [15]	Turkey	RC	630	STEMI	56.7	81.0	During hospitalization	12.5	primary	143.3	IScr ≥0.3 mg/dL within 48 h
Cicek et al. 2015 [16]	Turkey	PC	645	STEMI	56.5	85.3	Max: 6	13.6	primary	233.3	IScr > 0.5 mg/dL or 25% within 48 h
Crimi et al. 2015 [34]	Italy	PC	1443	STE-ACS 44.9% NSTE-ACS 55.1%	–	–	Mean: 22.8 Median: 24	10.8	N/A	–	IScr ≥25%
Giacoppo et al. 2015 [24]	USA	PC	9512	ACS	62.5	72.3	Max: 12	12.7	primary	221.5	IScr ≥0.5 mg/dL or 25% within 72 h
Turan et al. 2015 [26]	Turkey	RC	312	NSTEMI	59	76.0	Mean: 38 Max: 40	9.6	elective	154.8	IScr > 0.5 mg/dl or 25% within 72 h
Centola et al. 2016 [35]	Italy	RC	402	STEMI	64.9	72.0	Median: 12	17.4	primary	257.4	IScr ≥0.5 mg/dl or 25% within72h
Farhan et al. 2016 [36]	Austria	PC	536	STEMI 35.5% NSTEMI 47.0% UA 17.5%	62.7	68.1	Mean: 94 Max: 108.9	9.5	N/A	255.4	RIFLE/AKIN criteria
Gungor et al. 2016 [25]	Turkey	RC	587	STEMI	56.0	66.1	Median: 12 Max: 24	21.8	primary	163.2	IScr ≥0.5 mg/dL or 25% within 72 h
Kuboyama et al. 2016 [27]	Japan	PC	247	STEMI	66.7	76.5	Mean: 31.2	27.1	primary	152.1	IScr ≥0.5 mg/dL or 25% within 72 h
Nakahashi et al. 2016 [20]	Japan	RC	577	STEMI	64.4	78.7	Max: 36	35.7	primary	168.5	IScr ≥0.5 mg/dL or 25% within 72 h
Park et al. 2016 [23]	Korea	PC	668	STEMI	61.3	77.2	Mean: 26.4	10.9	primary	–	IScr ≥0.5 mg/dL or 25% within 48 h
Park et al. 2016(2) [17]	Korea	PC	334	STEMI	62.7	79.3	During hospitalization	21.6	primary	159.8	IScr ≥0.5 mg/dL or 25% within 72 h

*CI-AKI* contrast-induced acute kidney injury, *USA* the United States, *PC* prospective, *RC* retrospective, *AMI* acute myocardial infarction, *IScr* increased serum creatinine, *PCI* percutaneous coronary intervention, *STEMI* ST-elevation myocardial infarction, *NSTEMI* non-ST-elevation myocardial infarction, *UA* unstable angina, *STE-ACS* ST-elevation acute coronary syndrome, *NSTE-ACS* non-ST-elevation acute coronary syndrome, RIFLE criteria: ISCr ≥150% from baseline or a decrease in the eGFR≥25% within 72 h; AKIN criteria: ISCr≥0.3 mg/dL or 1.5-times the baseline using sCr criteria, or < 0.5 mL/kg/h for ≥6 h using urine-output criteria, within 48 h; N/A, not available

### Quality of included studies

**Fig. 2:**
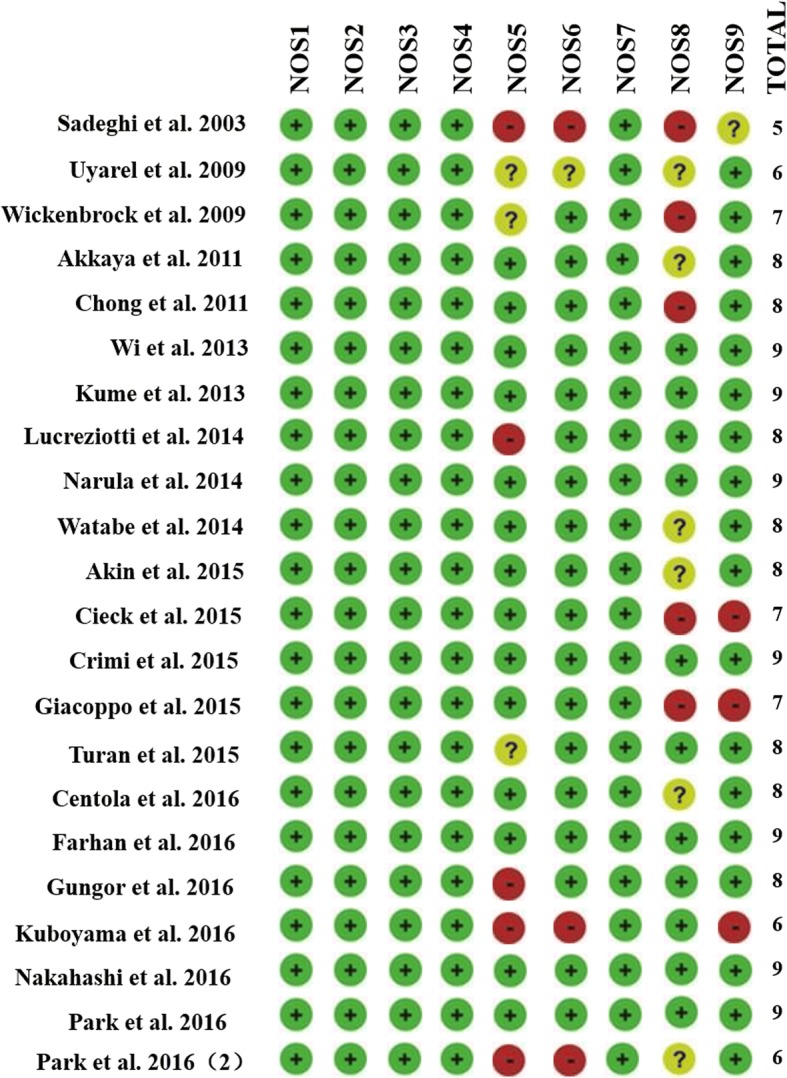
Assessment of study quality

The NOS scores for included studies were detailed in Fig. 2. Overall, 18 (81.8%) studies were regarded to be of good methodological quality and the remaining 4 studies [17, 27, 32, 37] were considered to be of fair quality.

### CI-AKI and risk of all-cause mortality

**Fig. 3:**
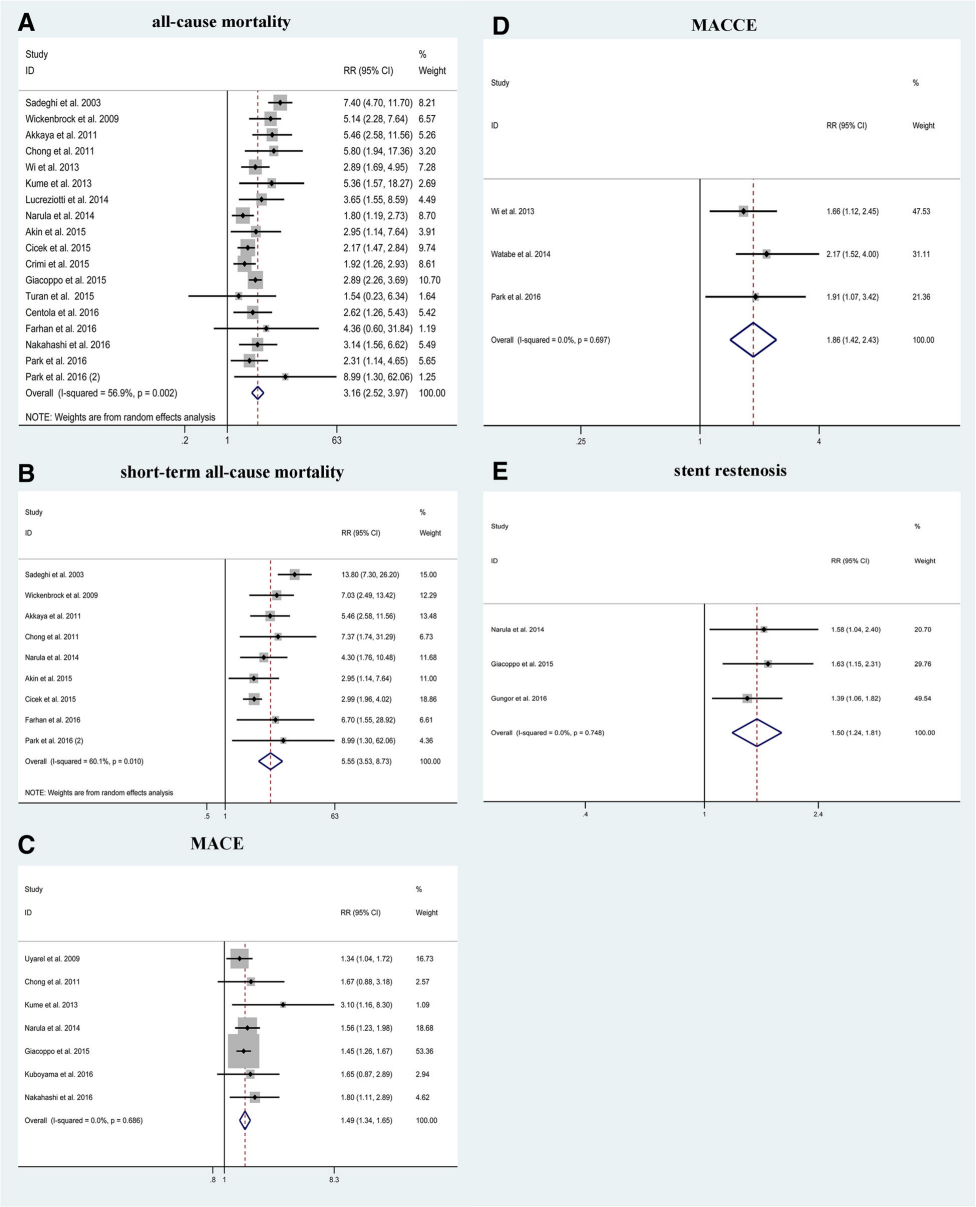
Association between contrast-induced acute kidney injury (CI-AKI) and risk of adverse clinical outcomes. **a** all-cause mortality, (**b**) short-term all-cause mortality, (**c**) major adverse cardiac events (MACE), (**d**) major adverse cardiovascular and cerebrovascular events (MACCE) and (**e**) stent restenosis. Abbreviations: RR, risk ratio

Of the 22 cohorts included, 18 studies (16 with adjusted RR [12–21, 23, 24, 26, 33–35] and 2 with crude RR [32, 36]) investigated the association between CI-AKI and risk of all-cause mortality for a total pooled population of 28,367 patients after PCI for ACS. Overall, CI-AKI was associated with a significantly increased risk of all-cause mortality (pooled RR 3.16; 95% CI 2.52–3.97; Q statistic *P* = 0.002; I^2^ = 56.9%; Fig. 3a). Notably, this data set was heterogeneous, thus random effects model was used for the analysis.

### CI-AKI and risk of short-term all-cause mortality

Of the 22 cohorts included, 7 studies investigated the association between CI-AKI and risk of in-hospital mortality [12, 13, 15–17, 19, 36], and 2 studies [32, 33] investigated the association between CI-AKI and risk of 30-day mortality for a total number of 13,895 patients after PCI for ACS. These 9 studies (7 with adjusted RRs [12, 13, 15–17, 19, 33] and 2 with crude RRs [32, 36]) were pool-evaluated using random-effects model with moderate heterogeneity, and revealed that CI-AKI was significantly associated with increased risk of short-term all-cause mortality (pooled RR 5.55; 95% CI 3.53–8.73; Q statistic *P* = 0.010; I^2^ = 60.1%; Fig. 3b).

### CI-AKI and risk of MACE, MACCE and stent restenosis

For ACS patients treated with PCI, CI-AKI increased the risk of MACE (7 studies [18–20, 24, 27, 33, 37]; *n* = 19,841; pooled RR 1.49, 95% CI: 1.34–1.65; Q statistic *P* = 0.686; I^2^ = 0; Fig. 3c), MACCE (3 studies [21–23]; *n* = 2768; pooled RR 1.86, 95% CI: 1.42–2.43; Q statistic *P* = 0.697; I^2^ = 0; Fig. 3d) and stent restenosis (3 studies [19, 24, 25]; *n* = 130,678; pooled RR 1.50, 95% CI: 1.24–1.81; Q statistic *P* = 0.748; I^2^ = 0; Fig. 3e), respectively. These data were not heterogeneous (all Q statistic *P* > 0.1 and all I^2^ = 0); therefore, fixed effects models were used for analyses.

### Subgroup analyses

**Fig. 4:**
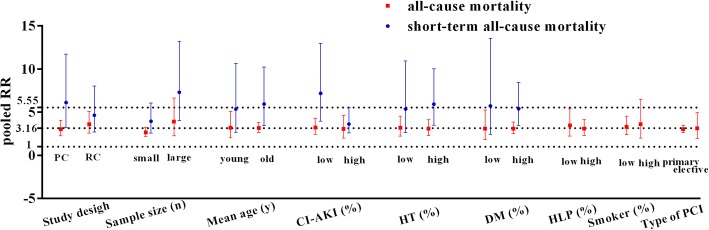
Subgroup and meta-regression analysis for all-cause mortality and short-term all-cause mortality. Abbreviations: RR, risk ratio; PC, prospective; RC, retrospective; CI-AKI, CI-AKI, contrast-induced acute kidney injury; HT, hypertension; DM, diabetes mellitus; HLP, hyperlipidemia; PCI, percutaneous coronary intervention. Hint: the cut points for all-cause mortality: sample size, 1500; mean age, 62 years old; prevalence of CI-AKI, 14.5%; prevalence of HT, 55%; prevalence of DM, 24.2%; prevalence of HLP, 48%; prevalence of smoker, 47.5% and for short-term all-cause mortality: sample size, 2000; mean age, 60 years old; prevalence of CI-AKI, 13.6%; prevalence of HT, 53%; prevalence of DM, 23.4%. The cut-off points are all the means for continuous data.

For all-cause mortality and short-term all-cause mortality, we further conducted subgroup meta-analysis. Figure 4 showed possible confounding factors and outcomes. In the subgroups of prospective study, sample size< 1500, mean age ≥ 62 years old, CI-AKI (%) ≥ 14.5, hypertension (HT) (%) ≥ 55, diabetes mellitus (DM) (%) ≥ 24.2 and hyperlipidemia (HLP) (%) ≥ 48 for all-cause mortality, and the subgroups of prospective study, sample size< 1500, sample size≥1500, mean age ≥ 60 years old, CI-AKI (%) < 13.6, CI-AKI (%) ≥ 13.6, HT (%) ≥ 53, DM (%) ≥ 23.4 and elective PCI for short-term all-cause mortality, no statistical heterogeneity was detected (all *P* values > 0.1). Even though all differences were not significant (all *P* > 0.05 in meta-regression analysis), an obvious difference of pooled RRs occurred in studies with different sample sizes for all-cause mortality, while obvious differences of pooled RRs were found in studies with different study design, sample size and prevalence of CI-AKI for short-term all-cause mortality (Fig. 4).

### Sensitivity analysis

The associations all remained significant after removing any single study conforming to Jackknife sensitivity analysis (see Additional files 5, 6, 7, 8 and 9).

### Publication Bias

There was no evidence of publication bias identified by Egger’s test for all adverse outcomes, with *P* = 0.205, 0.211, 0.053, 0.568 and 0.365 for all-cause mortality, short-term all-cause mortality, MACE, MACCE and stent restenosis, respectively (see Additional file 10).

## Discussion

To our knowledge, this study was the first meta-analysis to evaluate the association between CI-AKI and risk of adverse outcomes in ACS patients treated with PCI. Twenty two studies were eligible. We confirmed that CI-AKI might increase 216, 455, 49, 86 and 50% relative risk for all-cause mortality, short-term all-cause mortality, MACE, MACCE, and stent restenosis in ACS patients treated with PCI.

There was moderate heterogeneity among the studies included for all-cause mortality and short-term all-cause mortality, but no heterogeneity among the studies included for MACE, MACCE and stent restenosis.

CI-AKI was associated with an increased risk of all-cause mortality, short-term all-cause mortality, MACE, MACCE and stent restenosis of ACS patients after PCI in our study. Especially for short-term all-cause mortality, the increased risk was up to 455% compared to none CI-AKI patients. According to other reports, in ACS patients treated with PCI, CI-AKI was also associated with other adverse outcomes, including net adverse clinical events (HR = 1.53, 95% CI: 1.23–1.9 [19]), major bleeding (HR = 2.69, 95% CI: 2.26–3.2 [24] and HR = 2.07, 95% CI: 1.57–2.73 [19]) and in-hospital ischaemic stroke (HR = 2.91, 95% CI: 1.03–8.24 [38]).

The reasons for taking CI-AKI as a strong predictor of clinical adverse outcomes were complex. It was demonstrated that the renal function of 54.6% of CI-AKI patients did not return to baseline level after 2 weeks [39]. CI-AKI patients were at increased risk of progressive decline in kidney function [40, 41]. Over 45% of patients had persistent renal dysfunction leading to chronic kidney disease (CKD) [42]. Patients with CKD have more severe and diffuse coronary artery disease, higher rates of traditional coronary risk factors and complications such as diabetes mellitus, hypertension, left ventricular hypertrophy dyslipidemia, atrial fibrillation, and congestive heart failure [43, 44], and more restrictions to therapies for other diseases [43, 45].

For patients undergoing coronary angiography, CI-AKI was also reported to be associated with an increased risk of adverse clinical outcomes in a meta-analysis [46]. It included 39 studies and showed that CI-AKI was associated with increased risk of mortality (adjusted pooled RR = 2.39, 95% CI: 1.98–2.90), MACE (adjusted pooled RR = 1.98, 95% CI: 1.52–2.59) and end-stage renal disease (pooled crude RR, 15.26, 95% CI: 1.86–125.01) for patients treated with coronary angiography. Our meta-analysis focused on ACS patients treated with PCI. Noteworthy, only one same study [12] was included in both our study and the above study. The occurrence of ACS was more of an emergency and increased risk of short-term mortality associated with CI-AKI was higher (pooled RR 5.55; 95% CI 3.53–8.73). Therefore, CI-AKI should be paid more attention to for ACS patients treated with PCI.

There were some limitations of our meta-analysis. Firstly, the diagnostic criteria for CI-AKI, MACE and MACCE were not completely uniform in all included studies. Secondly, studies included in this meta-analysis were not adjusted for consistent factors when assessing the association between CI-AKI and outcomes. Finally, the differences of demographic and clinical characteristics regarding race, genetics, geographical locations and PCI procedure across the different populations pooled were not thoroughly studied. All the limitations might have effect on the risk evaluation of prognosis.

## Conclusions

Our review shows that CI-AKI is associated with increased adverse events. Hence, it will be useful in promptly detecting patients at high risk of CI-AKI, and thereby they can be carefully monitored and treated with adequate prophylactic strategy.

## Additional files

Additional file 1: PRISMA Checklist (PDF 353 kb)

Additional file 2: Search strategy to identify studies. (PDF 224 kb)

Additional file 3: Other related characteristics of included studies. (PDF 204 kb)

Additional file 4: Adjusted confounders in each included study. (PDF 243 kb)

Additional file 5: Sensitivity analysis for all-cause mortality. (PDF 106 kb)

Additional file 6: Sensitivity analysis for short-term all-cause mortality. (PDF 100 kb)

Additional file 7: Sensitivity analysis for major adverse cardiac events. (PDF 97 kb)

Additional file 8: Sensitivity analysis for major adverse cardiovascular and cerebrovascular events. (PDF 96 kb)

Additional file 9: Sensitivity analysis for stent restenosis. (PDF 92 kb)

Additional file 10: Egger’s test of all analysis. (PDF 186 kb)

## Abbreviations

ACS: Acute coronary syndrome
CI: Confidence intervals
CI-AKI: Contrast-induced acute kidney injury
CKD: Chronic kidney disease
DM: Diabetes mellitus
HLP: Hyperlipidemia
HR: Hazard ratio
HT: Hypertension
MACCE: Major adverse cardiovascular and cerebrovascular events
MACE: Major adverse cardiac events
NOS: Newcastle-Ottawa scale
NSTEMI: Non-ST elevation myocardial infarction
OR,: Odds ratio
PCI: Percutaneous coronary intervention
PRISMA: Preferred reporting items for systematic reviews and meta-analyses
RR: Risk ratio
STEMI: ST-elevation myocardial infarction
UA: Unstable angina
