# Supplementary material for: Contrast-induced acute kidney injury and adverse clinical outcomes risk in acute coronary syndrome patients undergoing percutaneous coronary intervention: a meta-analysis
Source: BMC Nephrol. 2018 Dec 22;19:374. doi: 10.1186/s12882-018-1161-5 (PMC6303898; doi:10.1186/s12882-018-1161-5)
Supplement: Supplementary file 2 — Search strategy to identify studies. (PDF 224 kb) [file 12882_2018_1161_MOESM2_ESM.pdf]

Additional file 2 Search strategy to identify studies

On Pubmed

Search (((((((((((((((((((mortality) OR cardiovascular) OR events) OR death) OR outcome) OR Hemodialysis) OR Haemodialysis) OR Dialysis) OR Adverse effect) OR prognosis) OR chronic kidney disease) OR CKD) OR end-stage renal disease) OR ESRD)) OR peritoneal dialysis)) AND (((((((((((acute myocardial infarction) OR AMI) OR myocardial infarction) OR MI) OR non-ST segment elevation myocardial infarction) OR ST-segment elevation myocardial infarction) OR acute coronary syndrome) OR ACS) OR unstable angina) OR acute ST-elevation myocardial infarction) OR acute non-ST segment elevation myocardial infarction)) AND ((((complete revascularization) OR revascularization) OR percutaneous coronary intervention) OR PCI)) AND (((((contrast-induced acute kidney injury) OR contrast-induced nephropathy) OR contrast media use) OR CIN) OR CI-AKI)

On Web of science™

TOPIC:((((((((((((((((((((mortality) OR cardiovascular) OR events) OR death) OR outcome) OR Hemodialysis) OR Haemodialysis) OR Dialysis) OR Adverse effect) OR prognosis) OR chronic kidney disease) OR CKD) OR end-stage renal disease) OR ESRD)) OR peritoneal dialysis)) AND (((((((((((acute myocardial infarction) OR AMI) OR myocardial

infarction) OR MI) OR non-ST segment elevation myocardial infarction) OR ST-segment elevation myocardial infarction) OR acute coronary syndrome) OR ACS) OR unstable angina) OR acute ST-elevation myocardial infarction) OR acute non-ST segment elevation myocardial infarction)) AND ((((complete revascularization) OR revascularization) OR percutaneous coronary intervention) OR PCI)) AND (((((contrast-induced acute kidney injury) OR contrast-induced nephropathy) OR contrast media use) OR CIN) OR CI-AKI))

On Cochrance library

#1: acute myocardial infarction

#2: AMI

#3: myocardial infarction

#4: MI

#5: non-ST segment elevation myocardial infarction

#6: ST-segment elevation myocardial infarction

#7: acute coronary syndrome

#8: ACS

#9: unstable angina

#10: acute ST-elevation myocardial infarction

#11: acute non-ST segment elevation myocardial infarction

#12: complete revascularization

#13: revascularization

#14: percutaneous coronary intervention

#15: PCI

#16: contrast-induced acute kidney injury

#17: contrast-induced nephropathy

#18: contrast media use

#19: CIN

#20: CI-AKI

#21: mortality

#22: cardiovascular

#23: events

#24: death

#25: outcome

#26: Hemodialysis

#27: Haemodialysis

#28: Dialysis

#29: peritoneal dialysis

#30: Adverse effect

#31: prognosis

#32: chronic kidney disease

#33: CKD

#34: end-stage renal disease

#35: ESRD

#36: #1 or #2 or #3 or #4 or #5 or #6 or #7 or #8 or #9 or #10 or #11 or #12 or #13 or #14 or #15

#37: #16 or #17 or #18 or #19 or #20

#38: #21 or #22 or #23 or #24 or #25 or #26 or # 27 or #28 or #29 or #30 or #31 or #32 or #33 or #34 or #35

#39: #36 and #37 and #38

We choose #39

We choose “trails”

On EMBASE

#1: acute myocardial infarction

#2: AMI

#3: myocardial infarction

#4: MI

#5: non-ST segment elevation myocardial infarction

#6: ST-segment elevation myocardial infarction

#7: acute coronary syndrome

#8: ACS

#9: unstable angina

#10: acute ST-elevation myocardial infarction

#11: acute non-ST segment elevation myocardial infarction

#12: complete revascularization

#13: revascularization

#14: percutaneous coronary intervention

#15: PCI

#16: contrast-induced acute kidney injury

#17: contrast-induced nephropathy

#18: contrast media

#19: CIN

#20: CI-AKI

#21: mortality

#22: cardiovascular

#23: events

#24: death

#25: outcome

#26: Hemodialysis

#27: Haemodialysis

#28: Dialysis

#29: peritoneal dialysis

#30: Adverse effect

#31: prognosis

#32: chronic kidney disease

#33: CKD

#34: end-stage renal disease

#35: ESRD

#36: #1 or #2 or #3 or #4 or #5 or #6 or #7 or #8 or #9 or #10 or #11 or #12 or #13 or #14 or #15

#37: #16 or #17 or #18 or #19 or #20

#38: #21 or #22 or #23 or #24 or #25 or #26 or # 27 or #28 or #29 or #30 or #31 or #32 or #33 or #34 or #35

#39: #36 and #37 and #38

We choose #39
