# Supplementary material for: Contrast-induced acute kidney injury and adverse clinical outcomes risk in acute coronary syndrome patients undergoing percutaneous coronary intervention: a meta-analysis
Source: BMC Nephrol. 2018 Dec 22;19:374. doi: 10.1186/s12882-018-1161-5 (PMC6303898; doi:10.1186/s12882-018-1161-5)
Supplement: Supplementary file 3 — Other related characteristics of included studies. (PDF 204 kb) [file 12882_2018_1161_MOESM3_ESM.pdf]

Additional file 3 Other related characteristics of included studies

Study	Baseline characteristic					Complication (%)				History (%)				Medication (%)			
	BMI (kg/m ^{2})	LVEF (%)	Scr (mg/dl)	eGFR (ml/min/1.73 m ^{2})	Killip class ≥2,%	HT	DM	HLP	Smoker	MI	P-CBS	P-PCI	Family CAD	Aspirin	ACEI/ARB	β-blocker	Statins
Sadeghi et al. 2003	-	-	-	-	10.6	48.0	16.9	38.0	43.3	13.3	1.9	-	-	99.0	45.5	87.4	31.4
Uyarel et al. 2009	-	47.4	0.97	-	6.1	39.3	24.6	34.5	57.9	10.6	2.9	8.0	16.2				
Wickenbrock et al. 2009	-	56.0	0.95	-	-	82.7	24.5	77.6	40.8	-	9.7	-	-				
Akkaya et al. 2010	-	-	0.97	-	6.0	39.5	24.2	36.7	62.2	11.8	2.9	-	17.5				
Chong et al. 2011	-	48.3	-	-	-	58.8	34.5	76.3	52.5	-	-	-	10.8				
Wi et al. 2012	24.1	48.4	1.06	-	-	49.0	26.0	38.0	38.0	6.0	2.0	13.0	-	97.0	84.0	80.0	87.0
Kume et al. 2013	23.9	-	0.86	73.7	15.5	73.2	33.0	68.0	-	5.2	-	-		13.4	21.6	9.3	16.5
Lucreziotti et al. 2014	26.1	-	1.00	76.7	13.0	52.3	17.0	33.1	42.7	-	-	-	17.0				
Narula et al. 2014	27.0	-	-	-	8.8	53.1	16.4	42.7	64.1	10.7	2.9	10.8	30.6	97.7	81.5	89.7	94.2
Watabe et al. 2014	24.0	55.6	-	68.1	14.3	62.0	34.4	49.4	56.8	10.5	1.7	11.7	-	23.9	29.2	16.1	17.0
Akin et al. 2015	-	49.1	0.98	94.4	-	36.8	25.7	-	36.5	18.9	3.5	-	-				
Cicek et al. 2015	-	45.0	0.90	-	6.4	41.3	19.7	-	-	-	-	-	-				
Crimi et al. 2015	26.6	53.7	1.00	79.1	-	71.7	24.2	54.0	24.1	26.3	10.5	18.2	27.9				
Giacoppo et al. 2015	-	61.1		64.9	-	61.5	24.3	51.6	35.0	24.2	13.1	30.2	-	98.2	39.7	62.9	44.8
Turan et al. 2015	28.3	52.0	1.04	-	4.5	62.5	29.6	51.8	43.2	-	10.6	14.1	-				
Centola et al. 2016	26.1	-	1.00	115	13	53	16	33	44.0	--	-	-	-				
Farhan et al. 2016	-	-	1.07	-	-	70.3	22.6	73.7	-	9.9	-	-	-		59.3		
Gungor et al. 2016	-	46.8	0.88	-	-	41.4	27.1	34.2	43.4	5.8	-	7.8	-				
Kuboyama et al. 2016	-		0.87	73.8	-	66.8	36.8	64.4	70.5	-	-	-	24.3				

Nakahashi et al. 2016	24.4	-	0.88	70.9	9.5	56.2	25.8	60.1	71.2	2.1	-	-	-	99.5	83.5	66.9	69.8
Park et al. 2016	-	45.3	1.06	-	25.9	51.6	30.1	24.7	-	-	-	-	-				
Park et al. 2016（2）	24.4	52.8	0.84	-	-	46.4	25.7	6.3	68.0	-	-	-	-	100		85.6	

Abbreviations: BMI=body mass index; LVEF=left ventricular ejection fraction; Scr= serum creatinine; eGFR= estimated glomerular filtration rate; HT=hypertension; DM=diabetes mellitus; HLP=Hyperlipidemia; MI=myocardial infarction; P-PCS= Previous coronary bypass surgery; P-PCI= Previous percutaneous coronary intervention; CAD=coronary artery disease.
