# Supplementary material for: Contrast-induced acute kidney injury and adverse clinical outcomes risk in acute coronary syndrome patients undergoing percutaneous coronary intervention: a meta-analysis
Source: BMC Nephrol. 2018 Dec 22;19:374. doi: 10.1186/s12882-018-1161-5 (PMC6303898; doi:10.1186/s12882-018-1161-5)
Supplement: Supplementary file 4 — Adjusted confounders in each included study. (PDF 243 kb) [file 12882_2018_1161_MOESM4_ESM.pdf]

Additional file 4 Adjusted confounders in each included study

Study	Adjusted confounders
Sadeghi et al. 2003	No adjusted confounders (for all-cause mortality and short-term all-cause mortality)
Uyarel et al. 2009	There were confounders but they were not clearly written (for MACE). We wrote to the corresponding author on September 24th but did not get a response.
Wickenbrock et al. 2009	The adjusted confounders were not explicitly stated in the text. But it was written that a multivariate regression model was applied including all the potential confounding variables leading to death. It was just shown the remained significant independent correlates of death in multivariate analysis including cardiogenic shock and highest total creatinkinasis (for all-cause mortality and short-term all-cause mortality). We wrote to the corresponding author on September 24th but did not get a response.
Akkaya et al. 2011	diabetes mellitus, myocardial infarction history, Killip class 2-3, use of Tirofiban, chronic kidney disease, female gender, failed operation, age> 70, multivessel disease, anemia during application (for all-cause mortality and short-term all-cause mortality)
Chong et al. 2011	age (>70years), gender (female), diabetes, anemia (haemoglobin < 11g/dL), baseline renal impairment (for all-cause mortality, short-term all-cause mortality and MACE)
Wi et al. 2012	previous stroke, body mass index<24 kg/m^2, serum creatinine>1.5 mg/dL, intra-aortic balloon pump, shock anemia, left ventricular ejection fraction<40%, age>75 years, female gender, hypertension (for all-cause mortality); all confounders above and multivessel disease (for MACCE)
Kume et al. 2013	It was only given the significantly adjusted confounders included age>75 years, anemia and previous myocardial infarction (for all-cause mortality); it was adjusted some confounders but not showed (for MACE). We wrote to the corresponding author on September 24th but did not get a response.
Lucreziotti et al. 2014	Mehran risk score class, left ventricular ejection fraction <40%, in-hospital major bleeding and female gender (for all-cause mortality)

Narula et al. 2014	white blood cell count, admission haemoglobin, ejection fraction, Killip class 2–4, history of prior myocardial infarction, age, baseline creatinine (for all-cause mortality); no adjusted confounders (for short-term all-cause mortality); admission haemoglobin, ejection fraction, age, platelet count, history of smoking, diabetes mellitus, left anterior descending artery disease, baseline creatinine and history of prior PCI (for MACE)
Watabe et al. 2014	age, female gender, peak creatinine kinase>4000, intra-aortic balloon pump use, chronic kidney disease, killip class≥2, acute ST-segment elevation myocardial infarction, left ventricular ejection fraction≤40%, peak creatine kinase >4000 and prior congestive heart failure (for MACCE)
Akin et al. 2015	age, hypertension, left ventricular ejection fraction, estimated glomerular filtration rate, neutrophil/lymphocyte ratio, red cell distribution width and multivessel coronary disease (for all-cause mortality and short-term all-cause mortality)
Cicek et al. 2015	age, male, diabetes mellitus, smoking, Killip class>1, ejection fraction, V/GFR, low-density lipoprotein, multi vessel disease, hemoglobin (for all-cause mortality and short-term all-cause mortality)
Crimi et al. 2015	age, gender, weight, height, diabetes mellitus, baseline serum creatinine, left ventricular ejection fraction, cardiogenic shock, peripheral artery disease, previous myocardial infarction, multivessel coronary artery disease and diagnosis (stable vs. ACS), DAPT duration, and stent type (for all-cause mortality)
Giacoppo et al. 2015	age, sex, diabetes, creatinine clearance <60 mL/min, contrast volume (per 100 mL increase), randomization to bivalirudin, randomization to glycoprotein IIb/IIIa inhibitor history of hypertension, hyperlipidemia, anemia, previous percutaneous coronary intervention, and previous coronary artery bypass grafting (for all-cause mortality, MACE and stent restenosis)
Turan et al. 2015	age, sex, diabetes, hypertension, smoking, previous coronary artery bypass grafting, previous PCI, cholesterol (total, low-density lipoprotein and high-density lipoprotein), duration of chest pain, peak troponin level, ST segment depression, left bundle branch block, left ventricular ejection fraction, presence of multivessel disease, baseline creatinine, creatinine at hospital discharge, creatinine clearance at hospital discharge, hemoglobin concentration (for all-cause mortality)

Centola et al. 2016	women, aged 75 years or older, smoking, body mass index, estimated glomerular filtration rate<60 ml/min/1.73 m^2, left ventricular ejection fraction<40%, in-hospital major bleedings, Mehran risk score>6, Killip class>2 (for all-cause mortality)
Farhan et al. 2016	No adjusted confounders (for all-cause mortality and short-term all-cause mortality)
Gungor et al. 2016	male gender, hypertension, presence of severe thrombus, stent length, glycoprotein IIb/IIIa receptor antagonist therapy, white blood cell (for stent restenosis)
Kuboyama et al. 2016	No adjusted confounders (for MACE)
Nakahashi et al. 2016	age, Killip class, and thrombolysis in myocardial infarction flow (for all-cause mortality and MACE)
Park et al. 2016	age, diabetes, hypertension, multi-vessel disease, Killip class 4, left ventricular ejection fraction, hypoxic liver injury (for all-cause mortality and MACCE)
Park et al. 2016（2）	No adjusted confounders (for all-cause mortality and short-term all-cause mortality)
