# Supplementary material for: Contrast-induced acute kidney injury and adverse clinical outcomes risk in acute coronary syndrome patients undergoing percutaneous coronary intervention: a meta-analysis
Source: BMC Nephrol. 2018 Dec 22;19:374. doi: 10.1186/s12882-018-1161-5 (PMC6303898; doi:10.1186/s12882-018-1161-5)
Supplement: Supplementary file 5 — Sensitivity analysis for all-cause mortality. (PDF 106 kb) [file 12882_2018_1161_MOESM5_ESM.pdf]

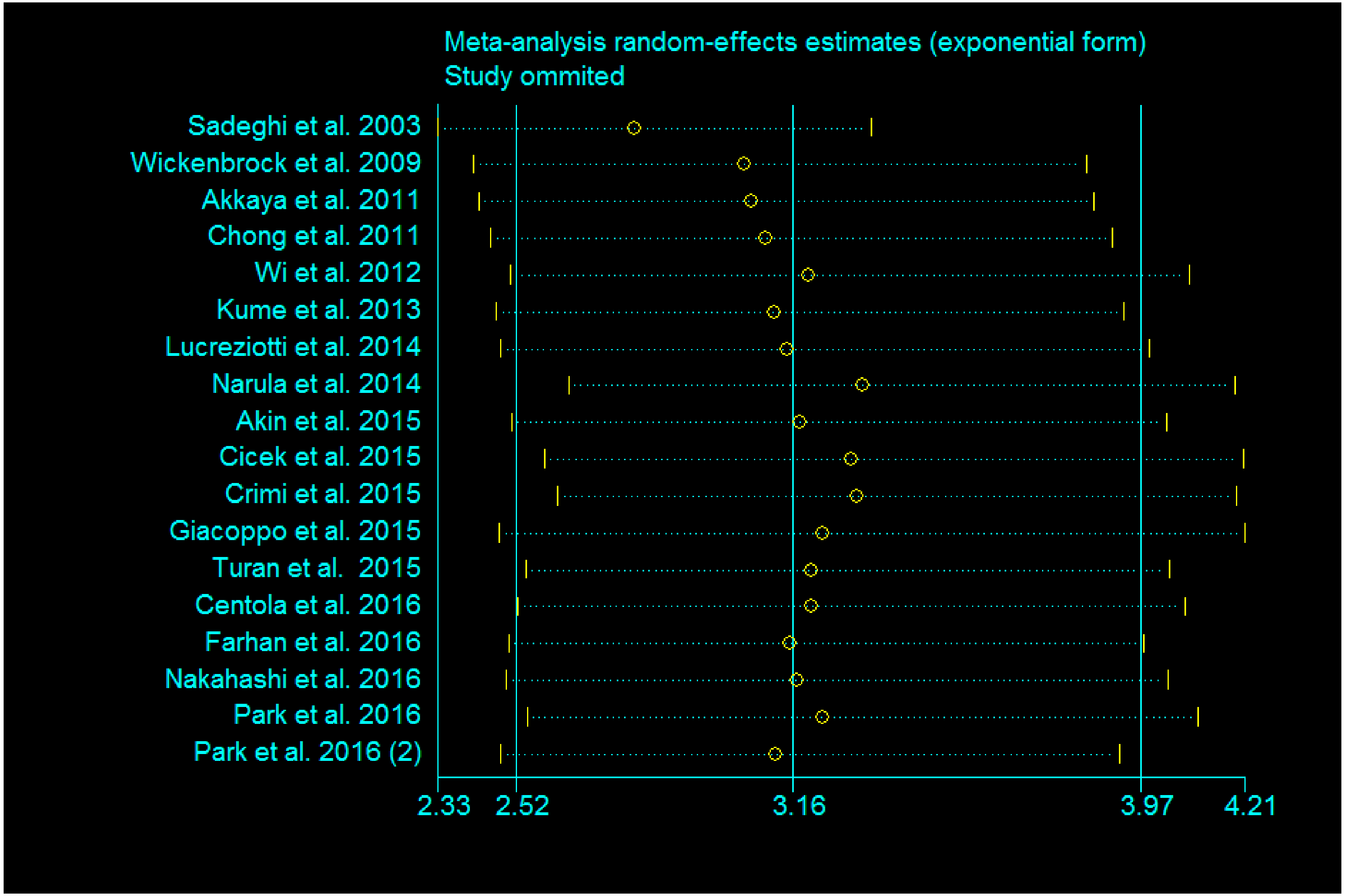
Meta-analysis random-effects estimates (exponential form)
Study ommited
Sadeghi et al. 2003
Wickenbrock et al. 2009
Akkaya et al. 2011
Chong et al. 2011
Wi et al. 2012
Kume et al. 2013
Lucreziotti et al. 2014
Narula et al. 2014
Akin et al. 2015
Cicek et al. 2015
Crimi et al. 2015
Giacoppo et al. 2015
Turan et al. 2015
Centola et al. 2016
Farhan et al. 2016
Nakahashi et al. 2016
Park et al. 2016
Park et al. 2016 (2)
2.33
2.52
3.16
3.97
4.21

Additional file 5: Sensitivity analysis for all-cause mortality.
