# Supplementary material for: Contrast-induced acute kidney injury and adverse clinical outcomes risk in acute coronary syndrome patients undergoing percutaneous coronary intervention: a meta-analysis
Source: BMC Nephrol. 2018 Dec 22;19:374. doi: 10.1186/s12882-018-1161-5 (PMC6303898; doi:10.1186/s12882-018-1161-5)
Supplement: Supplementary file 6 — Sensitivity analysis for short-term all-cause mortality. (PDF 100 kb) [file 12882_2018_1161_MOESM6_ESM.pdf]

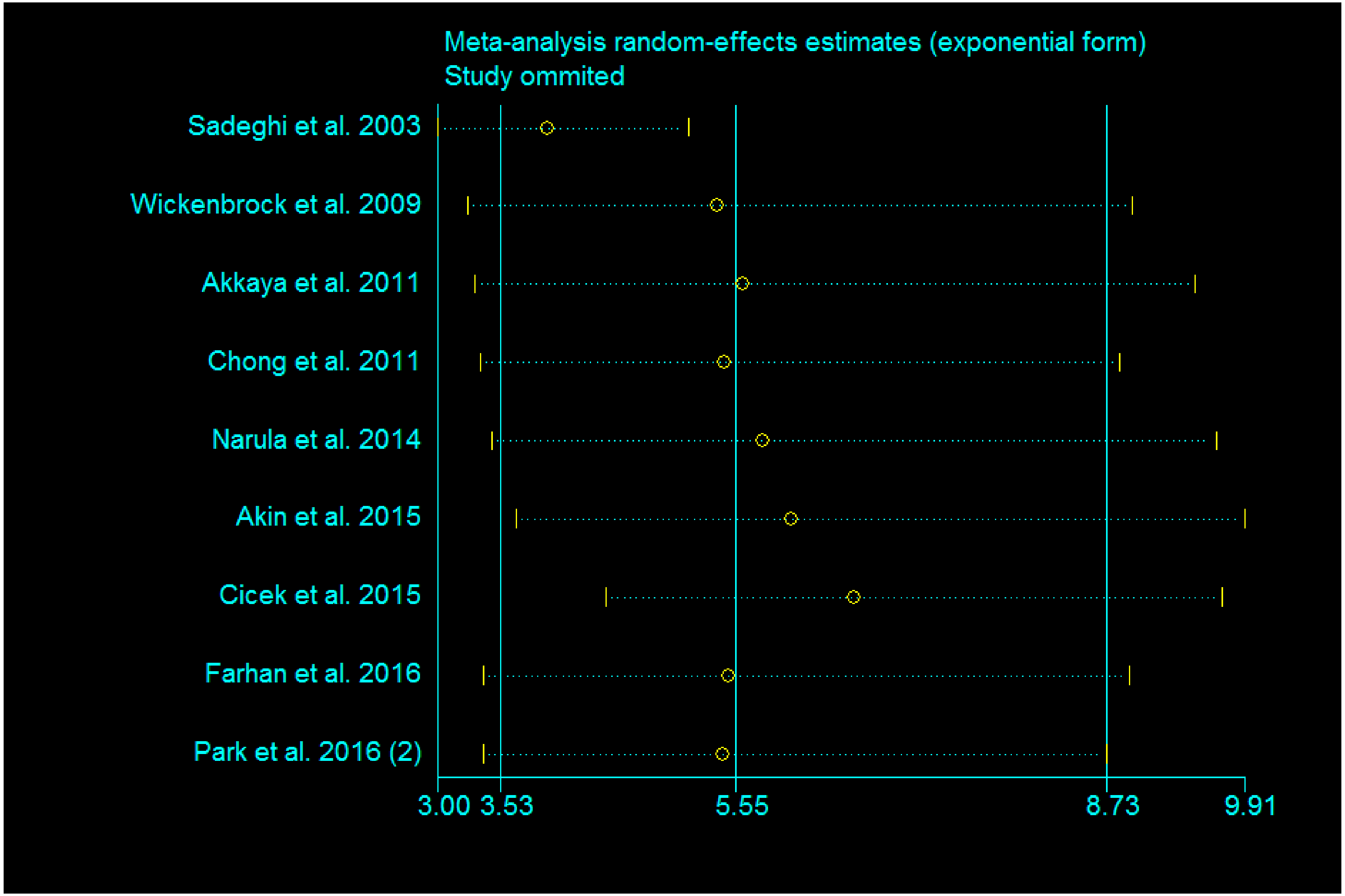
Meta-analysis random-effects estimates (exponential form)
Study ommited
Sadeghi et al. 2003
Wickenbrock et al. 2009
Akkaya et al. 2011
Chong et al. 2011
Narula et al. 2014
Akin et al. 2015
Cicek et al. 2015
Farhan et al. 2016
Park et al. 2016 (2)
3.00
3.53
5.55
8.73
9.91

Additional file 6: Sensitivity analysis for short-term all-cause mortality.
