# Supplementary material for: Contrast-induced acute kidney injury and adverse clinical outcomes risk in acute coronary syndrome patients undergoing percutaneous coronary intervention: a meta-analysis
Source: BMC Nephrol. 2018 Dec 22;19:374. doi: 10.1186/s12882-018-1161-5 (PMC6303898; doi:10.1186/s12882-018-1161-5)
Supplement: Supplementary file 7 — Sensitivity analysis for major adverse cardiac events. (PDF 97 kb) [file 12882_2018_1161_MOESM7_ESM.pdf]

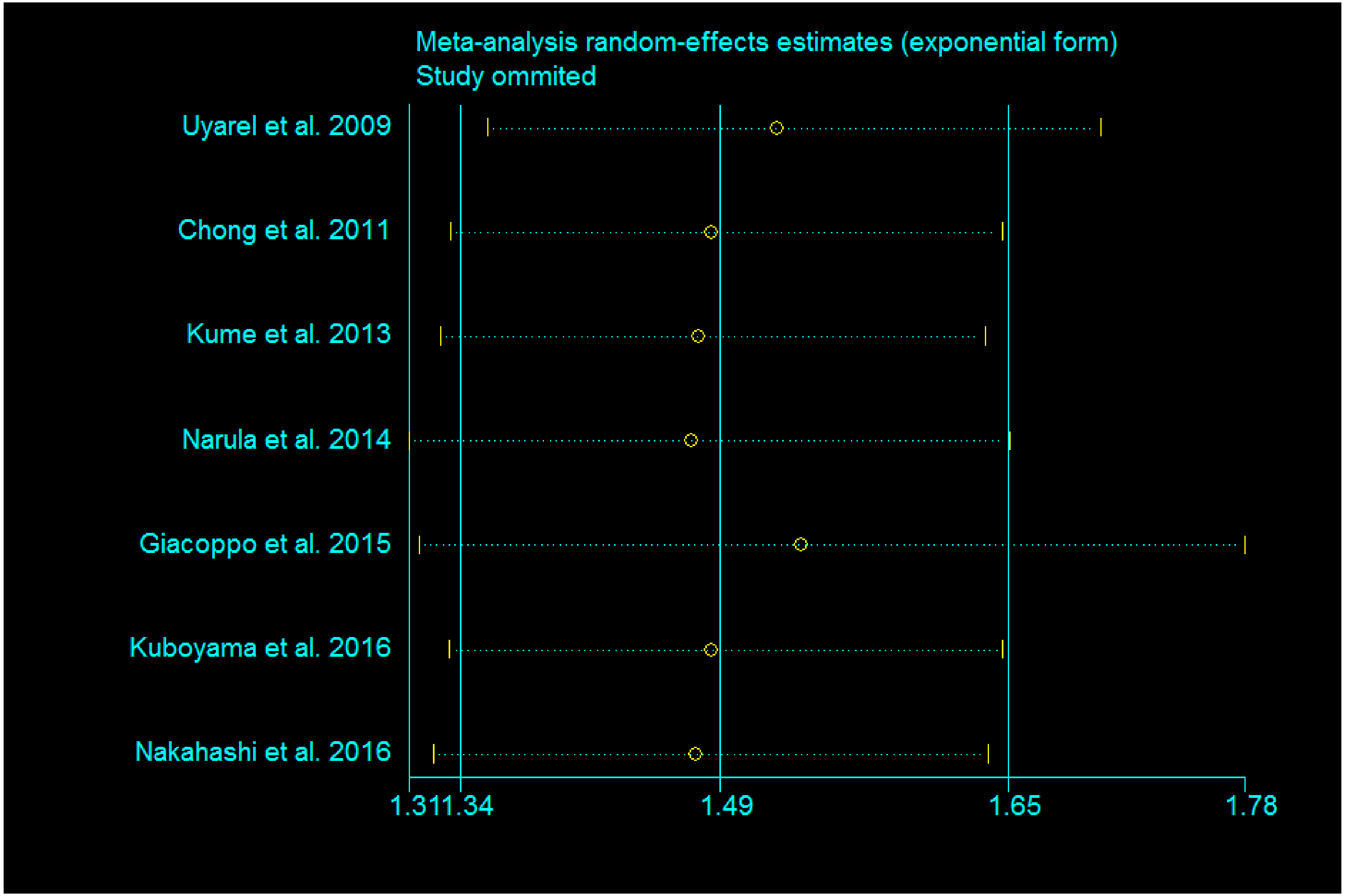
Meta-analysis random-effects estimates (exponential form)
Study ommited
Uyarel et al. 2009
Chong et al. 2011
Kume et al. 2013
Narula et al. 2014
Giacoppo et al. 2015
Kuboyama et al. 2016
Nakahashi et al. 2016
1.31
1.34
1.49
1.65
1.78

Additional file 7: Sensitivity analysis for major adverse cardiac events.
