# Supplementary material for: Contrast-induced acute kidney injury and adverse clinical outcomes risk in acute coronary syndrome patients undergoing percutaneous coronary intervention: a meta-analysis
Source: BMC Nephrol. 2018 Dec 22;19:374. doi: 10.1186/s12882-018-1161-5 (PMC6303898; doi:10.1186/s12882-018-1161-5)
Supplement: Supplementary file 8 — Sensitivity analysis for major adverse cardiovascular and cerebrovascular events. (PDF 96 kb) [file 12882_2018_1161_MOESM8_ESM.pdf]

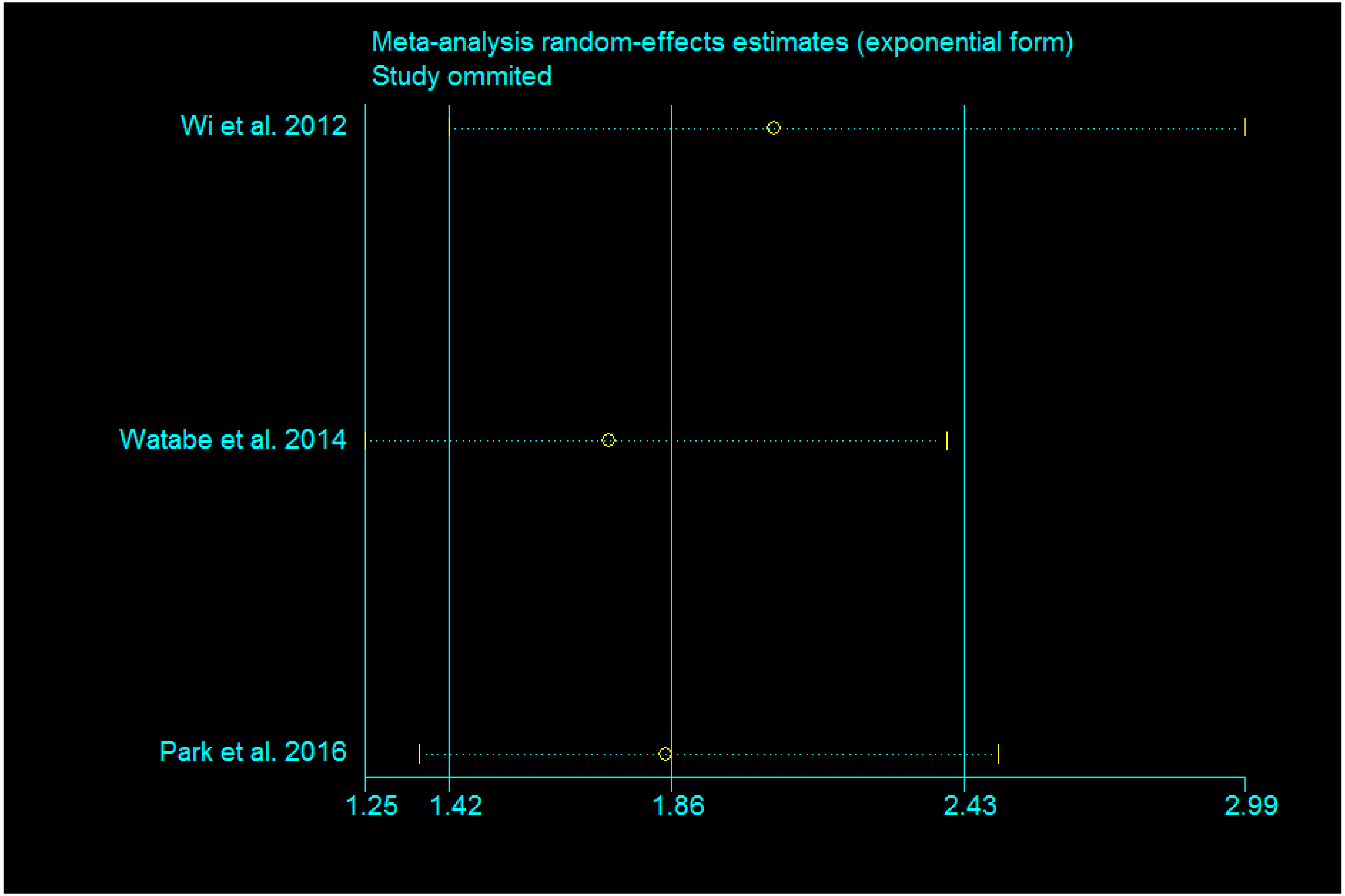
Meta-analysis random-effects estimates (exponential form)
Study ommited
Wi et al. 2012
Watabe et al. 2014
Park et al. 2016
1.25
1.42
1.86
2.43
2.99

Additional file 8: Sensitivity analysis for major adverse cardiovascular and cerebrovascular events.
