# Supplementary material for: Contrast-induced acute kidney injury and adverse clinical outcomes risk in acute coronary syndrome patients undergoing percutaneous coronary intervention: a meta-analysis
Source: BMC Nephrol. 2018 Dec 22;19:374. doi: 10.1186/s12882-018-1161-5 (PMC6303898; doi:10.1186/s12882-018-1161-5)
Supplement: Supplementary file 9 — Sensitivity analysis for stent restenosis. (PDF 92 kb) [file 12882_2018_1161_MOESM9_ESM.pdf]

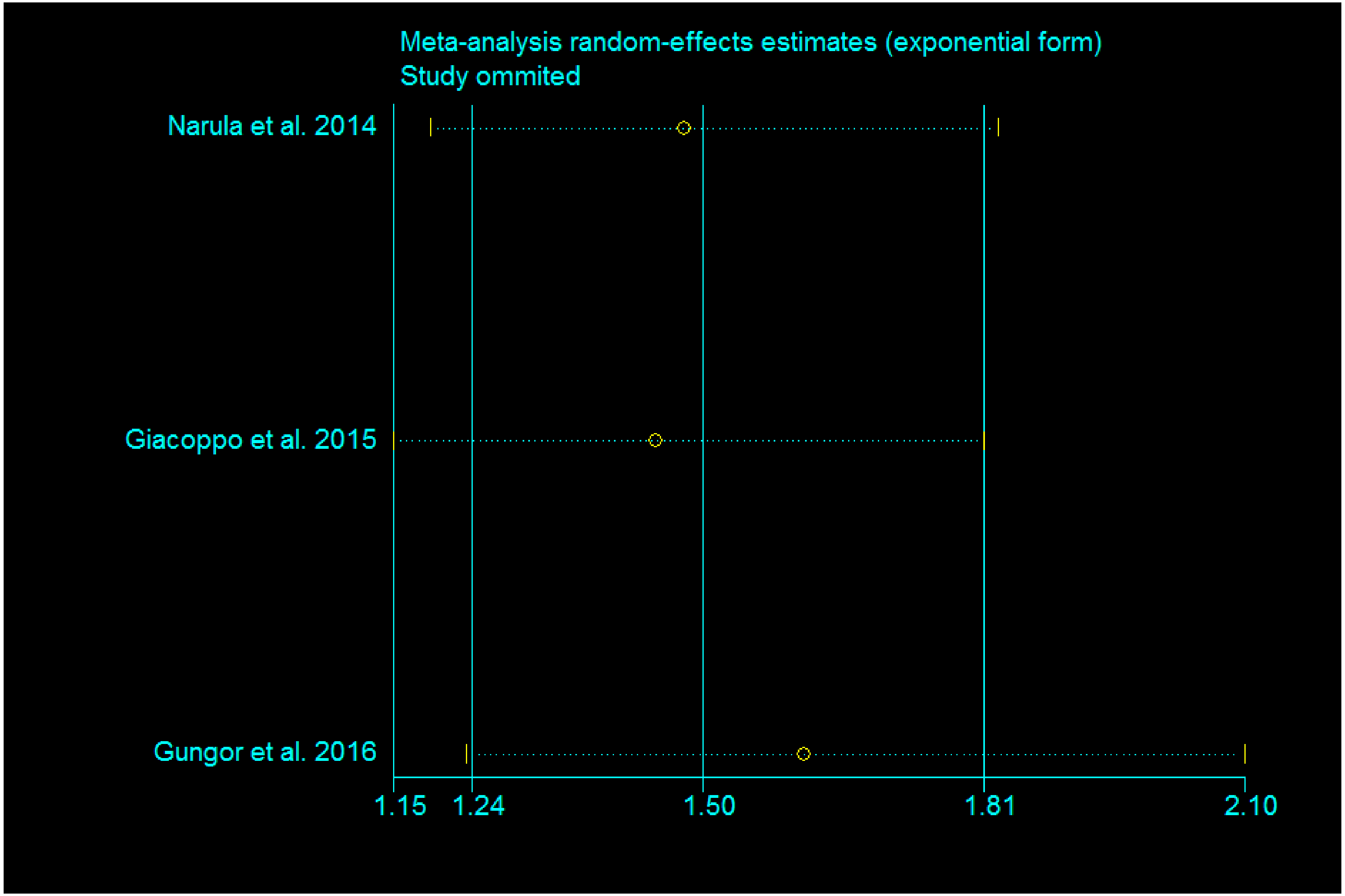

Meta-analysis random-effects estimates (exponential form)
Study ommited
Narula et al. 2014
Giacoppo et al. 2015
Gungor et al. 2016
1.15
1.24
1.50
1.81
2.10

Additional file 9: Sensitivity analysis for stent restenosis.
