# Supplementary material for: Contrast-induced acute kidney injury and adverse clinical outcomes risk in acute coronary syndrome patients undergoing percutaneous coronary intervention: a meta-analysis
Source: BMC Nephrol. 2018 Dec 22;19:374. doi: 10.1186/s12882-018-1161-5 (PMC6303898; doi:10.1186/s12882-018-1161-5)
Supplement: Supplementary file 10 — Egger’s test of all analysis. (PDF 186 kb) [file 12882_2018_1161_MOESM10_ESM.pdf]

Additional file 10: Egger's test of all analysis

All-cause mortality

Egger's test

Std_Eff	Coef.	Std. Err.	t	P>|t|	[95% Conf.	Interval]
slope	.8457414	.1961945	4.31	0.001	.4298277	1.261655
bias	.9336245	.706343	1.32	0.205	-.5637557	2.431005

Short-term all-cause mortality

Egger's test

Std_Eff	Coef.	Std. Err.	t	P>|t|	[95% Conf.	Interval]
slope	1.054995	.3991826	2.64	0.033	.1110784	1.998912
bias	1.478846	1.07418	1.38	0.211	-1.061187	4.01888

Major adverse cardiac events

gger's test

Std_Eff	Coef.	Std. Err.	t	P>|t|	[95% Conf.	Interval]
slope	.2769561	.0568737	4.87	0.005	.1307575	.4231547
bias	1.03511	.4094685	2.53	0.053	-.0174624	2.087682

Major adverse cardiovascular and cerebrovascular events

gger's test

Std_Eff	Coef.	Std. Err.	t	P>|t|	[95% Conf.	Interval]
slope	.170633	.5643209	0.30	0.813	-6.999743	7.341009
bias	1.909615	2.366636	0.81	0.568	-28.16134	31.98058

Stent restenosis

Egger's test

Std_Eff	Coef.	Std. Err.	t	P>|t|	[95% Conf.	Interval]
slope	.0595918	.2254548	0.26	0.835	-2.805083	2.924266
bias	2.077205	1.341045	1.55	0.365	-14.96239	19.1168
